# QM/MM Simulations Reveal the Determinants of Carbapenemase Activity in Class A β-Lactamases

**DOI:** 10.1021/acsinfecdis.2c00152

**Published:** 2022-07-25

**Authors:** Ewa I. Chudyk, Michael Beer, Michael A. L. Limb, Charlotte A. Jones, James Spencer, Marc W. van der Kamp, Adrian J. Mulholland

**Affiliations:** † Centre for Computational Chemistry, School of Chemistry, University of Bristol, Cantock’s Close, Bristol BS8 1TS, United Kingdom; ‡ School of Biochemistry, University of Bristol Medical Sciences Building, University Walk, Bristol BS8 1TD, United Kingdom; § School of Cellular and Molecular Medicine, University of Bristol Medical Sciences Building, University Walk, Bristol BS8 1TD, United Kingdom

**Keywords:** antibiotic resistance, carbapenem, computational enzymology, umbrella sampling, electrostatic stabilization

## Abstract

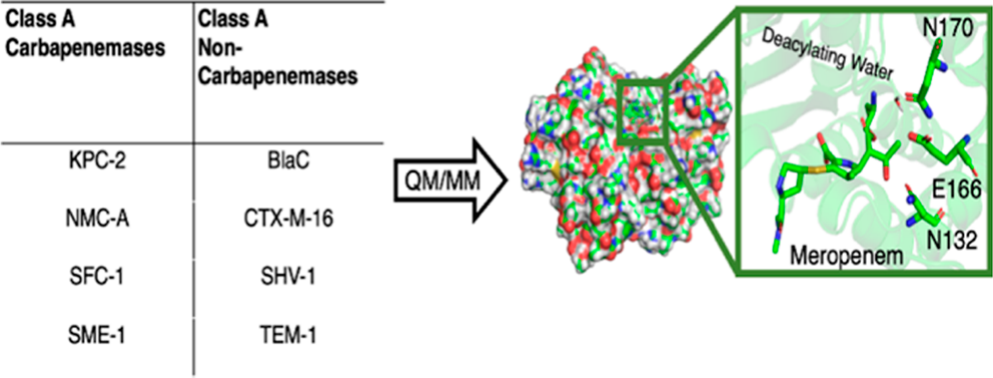

β-lactam antibiotic resistance in Gram-negative bacteria, primarily caused by β-lactamase enzymes that hydrolyze the β-lactam ring, has become a serious clinical problem. Carbapenems were formerly considered “last resort” antibiotics because they escaped breakdown by most β-lactamases, due to slow deacylation of the acyl-enzyme intermediate. However, an increasing number of Gram-negative bacteria now produce β-lactamases with carbapenemase activity: these efficiently hydrolyze the carbapenem β-lactam ring, severely limiting the treatment of some bacterial infections. Here, we use quantum mechanics/molecular mechanics (QM/MM) simulations of the deacylation reactions of acyl-enzyme complexes of eight β-lactamases of class A (the most widely distributed β-lactamase group) with the carbapenem meropenem to investigate differences between those inhibited by carbapenems (TEM-1, SHV-1, BlaC, and CTX-M-16) and those that hydrolyze them (SFC-1, KPC-2, NMC-A, and SME-1). QM/MM molecular dynamics simulations confirm the two enzyme groups to differ in the preferred acyl-enzyme orientation: carbapenem-inhibited enzymes favor hydrogen bonding of the carbapenem hydroxyethyl group to deacylating water (DW). QM/MM simulations of deacylation give activation free energies in good agreement with experimental hydrolysis rates, correctly distinguishing carbapenemases. For the carbapenem-inhibited enzymes, free energies for deacylation are significantly higher than for the carbapenemases, even when the hydroxyethyl group was restrained to prevent interaction with the DW. Analysis of these simulations, and additional simulations of mutant enzymes, shows how factors including the hydroxyethyl orientation, the active site volume, and architecture (conformations of Asn170 and Asn132; organization of the oxyanion hole; and the Cys69-Cys238 disulfide bond) collectively determine catalytic efficiency toward carbapenems.

Antibiotic resistance is a serious medical problem on all continents, affecting healthcare systems and economies.^1−4^ A particular threat is the rapid global increase in infections caused by Gram-negative bacteria, such as *Enterobacterales* (including *Escherichia coli* and *Klebsiella pneumoniae*) and *Pseudomonas aeruginosa*.^5−7^ An important contributory factor is the activity of class A β-lactamases,^8^ enzymes that can destroy the β-lactam rings of several classes of β-lactam antibiotics, such as penicillins (e.g. benzylpenicillin, Chart 1B), cephalosporins and carbapenems (e.g. meropenem, Chart 1A).^9−12^ In recent years, even the carbapenems, previously known as last-resort β-lactam antibiotics, because of lack of resistance, have become susceptible to β-lactamases.^13−15^ This threatens treatment for many bacterial infections.^16−18^ Such is the seriousness of this threat that the U.S. Centers for Disease Control and Prevention (CDC) have classified carbapenem-resistant *Enterobacterales* as Urgent Antimicrobial Resistance Threat pathogens.^19^

**Chart 1:**
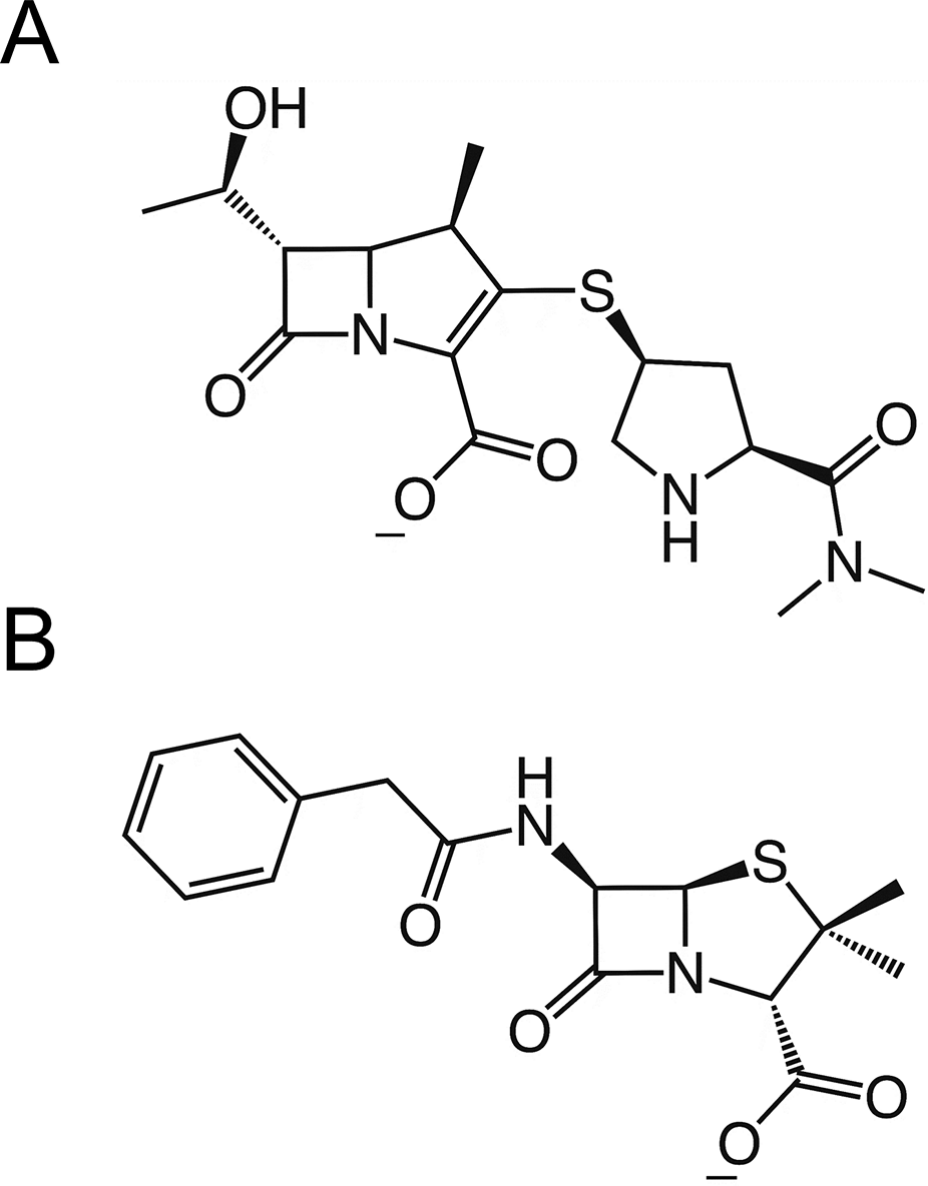
Structures of Antibiotics Used in This Study, Meropenem (A) and Benzylpenicillin (B)

β-Lactamases are divided into four classes by the Ambler classification system based on sequence similarity: serine β-lactamases of classes A, C, and D and class B, in which zinc ion(s) are involved in catalysis.^20,21^ Serine β-lactamases are believed to have evolved from the main targets of β-lactam antibiotics, the penicillin-binding proteins (PBPs), which catalyze the transpeptidation step in bacterial cell wall synthesis.^22,23^ PBPs are inhibited by β-lactams through the formation of a stable acyl-enzyme.^23^ In class A, C and D β-lactamases, however, deacylation of the acyl-enzyme can occur efficiently, leading to the release of the hydrolyzed β-lactam ring, preventing PBP inhibition. Class A enzymes, including the TEM, CTX-M, and KPC families, are currently the most widespread β-lactamases in the clinic.^22−28^

The β-lactam hydrolysis mechanism starts with acylation which, in class A enzymes, occurs through a nucleophilic attack of the active site Ser70 on the carbonyl carbon of the β-lactam ring. In carbapenem-inhibited class A β-lactamases, including TEM-1, SHV-1, BlaC, and CTX-M-16, the formation of a long-lasting carbapenem acyl-enzyme complex (Scheme 1A) is the reason for inhibition: the deacylation rate in these enzymes is low.^29,30^ In contrast, enzymes such as KPC-2 (now widely distributed in *K. pneumoniae* in the clinic), SFC-1, NMC-A, and SME-1 have the ability to deacylate significantly faster and confer bacterial resistance through efficient deactivation of carbapenem antibiotics.

**Scheme 1:**
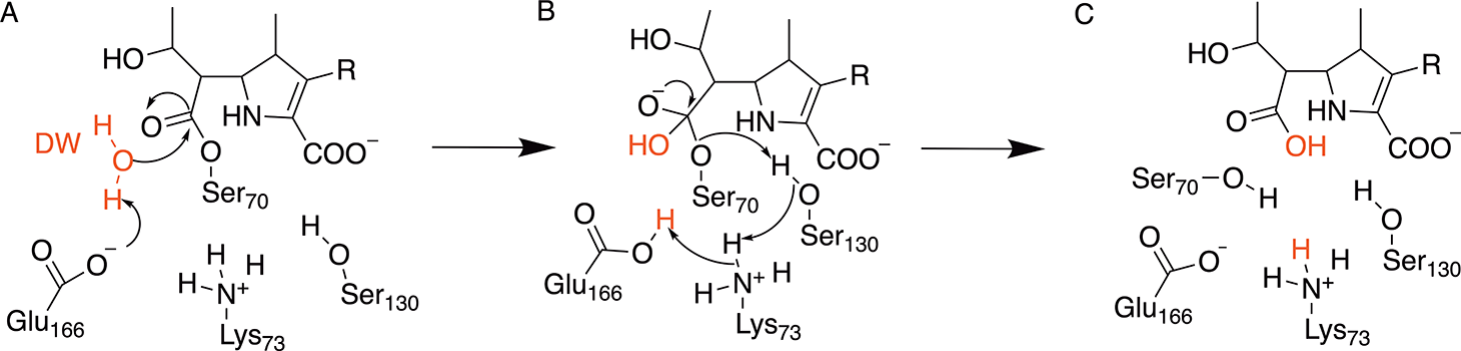
Proposed Carbapenem Deacylation Mechanism for Class A β-Lactamases;^31−34^ In the Acyl-enzyme, (A) Proton Transfer from Deacylating Water (DW, Red) to Glu166, with Subsequent Nucleophilic Attack on the Carbapenem Acylenzyme Carbonyl Carbon, Results in Formation of a Tetrahedral Intermediate (B). Collapse of this Oxyanion Gives the Hydrolyzed Carbapenem (C).

The deacylation mechanism (Scheme 1) is now widely accepted.^35,29^ The deacylating water (DW) plays a crucial role in the reaction by attacking the carbonyl carbon of the acyl-enzyme, assisted by abstraction of a proton by Glu166.^36^ Formation of the tetrahedral intermediate (TI) (Scheme 1B) is thought to be rate-limiting in deacylation.^37^ The collapse of the tetrahedral intermediate is accompanied by three proton transfers: transfer of a proton from Ser130 to the Ser70 side-chain oxygen with simultaneous transfers of protons from Lys73 to Ser130 and from Glu166 to Lys73 (Scheme 1B). This restores the resting state of the enzyme and the cleaved product, devoid of antibiotic activity, is released from the active site.

Whilst the rates of carbapenem deacylation differ significantly between carbapenem hydrolyzing and carbapenem inhibited Class A enzymes, crystal structures do not show large differences between enzymes with these different activities.^38,39^ The main structural differences within the class A β-lactamases (and their carbapenem acyl-enzymes) include the environment of the carbapenem 6α-1R-hydroxyethyl group and its interactions with the DW and Asn132; subtle shifts in the positions of active site residues such as Asn170, Ser130, and Ser70; changes in the distances between components of the oxyanion hole (i.e., the backbone amide groups of Ser70 and Ala/Ser/Thr237); and the presence/absence of a disulfide bridge between Cys69 and Cys238.^38^

Previously, we have shown that quantum mechanics/molecular mechanics (QM/MM) umbrella sampling simulations of the rate-limiting first step of deacylation (formation of the TI, Scheme 1B) can distinguish between carbapenemases and carbapenem-inhibited class A β-lactamases.^40,41^ In that work, meropenem breakdown efficiency is correctly captured using Glu166 as the general base for the deacylation reaction. Here, we use a similar approach in a detailed computational investigation into the origins of the differences in activity between the two groups of enzymes. We compare the carbapenemases KPC-2, SFC-1, SME-1, and NMC-A with the carbapenem-inhibited BlaC, TEM-1, SHV-1, and CTX-M-16 class A β-lactamases. We extend our simulations to several variants (including SFC-1 Asn132Gly, BlaC Gly132Asn, and SFC-1 Cys238Gly) to investigate the influence of specific interactions on deacylation activity. Establishing how subtle differences between enzymes able to destroy carbapenems and those that cannot affect turnover of carbapenems may guide the design of new β-lactam antibiotics that are more resistant to breakdown by currently circulating β-lactamase enzymes.

## Results

### Free Energy Reaction Path Calculations

**Figure 1:**
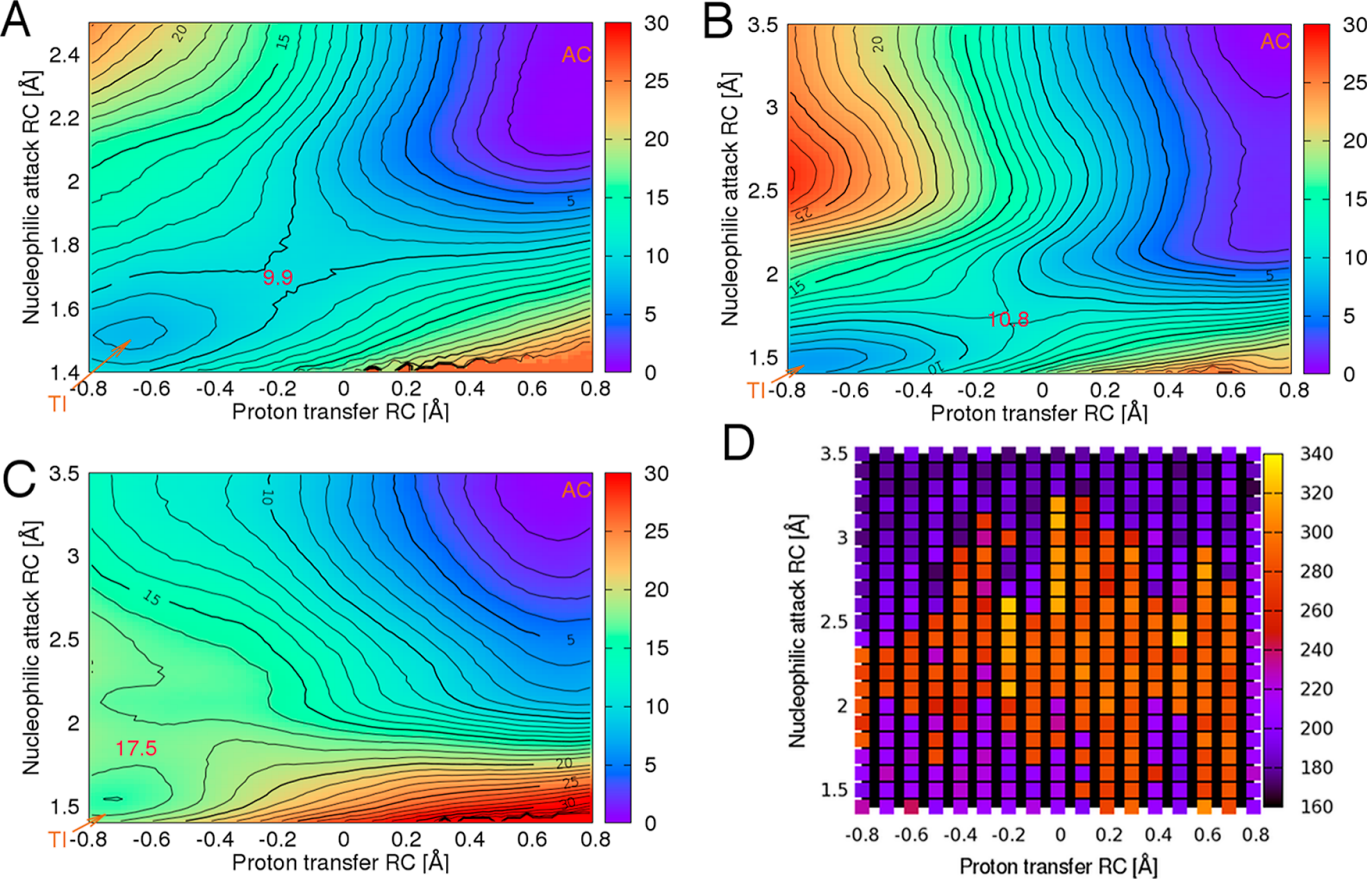
Free energy surfaces (SCC-DFTB/AMBER12SB QM/MM) for TI formation in the deacylation reaction of the acyl-enzymes (A) TEM-1/benzylpenicillin; (B) SFC-1/meropenem; and (C) TEM-1/meropenem. Energies are relative to the acyl-enzyme, in kcal/mol. AC, acyl-enzyme; TI, tetrahedral intermediate; and RC, reaction coordinate. The proton transfer reaction coordinate refers to the distance between the Glu166 side chain oxygen OE2 and the DW proton H2 minus the distance between the DW oxygen and DW proton H2, for example, in the TI, the proton transfer RC is 1 Å distance between Glu166 OE2 and DW proton H2 minus the 1.8 Å distance between DW oxygen and DW H2, resulting in a proton transfer RC of −0.8 Å. The nucleophilic attack RC is the distance between the DW oxygen and the carbonyl carbon of meropenem (Figure 2A, Chart S1). For the SFC-1/meropenem free energy surface, the corresponding final values for the hydroxyethyl dihedral angle (see Figure 3) are shown in (D).

For the 8 enzymes investigated, the differences in calculated activation free energy barriers for the first step of deacylation (acyl-enzyme modeled as Δ2 tautomer, see Figure 1) show good correspondence with differences in apparent barriers derived from published kinetic experiments (*k*_cat_ values, Table S1). Our previous results^40^ indicated that activation energy barriers for meropenem deacylation lower than around 13 kcal/mol are found for carbapenemases at this level of theory, while barriers above approximately 17 kcal/mol are calculated for carbapenem-inhibited enzymes. These calculated barrier heights are slightly underestimated compared to apparent experimental barriers (due to the SCC-DFTB method), but the discrimination between carbapenem-inhibited and carbapenem-hydrolyzing enzymes is well reproduced.^40,41^ This demonstrates that our simulation approach can serve as a starting point for the analysis of factors affecting energy barriers in the enzymes considered here.

The overall shapes of the free energy surfaces for the first step in carbapenem deacylation by all enzymes, except for TEM-1 with meropenem (Figure 1C), are similar, indicating that the reaction in the different enzymes proceeds in a similar manner. The SFC-1/meropenem free energy surface shown in Figure 1B is representative of those for all enzyme/meropenem complexes, other than that of TEM-1. The TEM-1/benzylpenicillin and TEM-1/meropenem surfaces are also shown due to their differing in the substrate or appearance of the free energy surface, respectively*.* Starting from the acyl-enzyme state (with reaction coordinate values for nucleophilic attack and for proton transfer of 3.5 and 0.8 Å, respectively), the reaction follows a minimum energy pathway toward the TI (reaction coordinate values for nucleophilic attack and proton transfer of 1.5 and −0.8 Å, respectively), reaching a transition state that is typically located at values around 1.7 and −0.2 Å, except TEM-1/meropenem at approximately 1.8 and −0.6 Å. The shape of those energy surfaces, with the transition state generally in the central part of the plot, indicates that the proton transfer and nucleophilic attack happen concertedly, rather than stepwise. (Of note, similar behavior is also observed for the reaction of TEM-1 with the good substrate benzylpenicillin, as shown in Figure 1A).

The QM/MM barrier differences and similarity in free energy surfaces suggest that the activity difference between carbapenemases and carbapenem-inhibited enzymes arises from differences in the interactions of the carbapenem with its immediate environment. The active site of class A β-lactamases is reasonably well conserved between different family members, with shared structural motifs (Figure S1). Aside from the catalytic residues Ser70 and Glu166, the active site components that are key for carbapenemase activity are the oxyanion hole (formed by the backbone amides of Ser70 and Ala/Ser/Thr237) and residues neighboring Glu166 and the DW (Lys73, Asn132, Asn170).^38,42^ In addition, interactions involving the 6-1R-hydroxyethyl group (i.e., part of the carbapenem core) may influence carbapenemase activity: it can form hydrogen bonds with active site residues as well as with the DW.^43,44^ In the following sections, we assess how these different factors affect the deacylation of the carbapenem acyl-enzyme complex.

### Role of the 6α-1R-Hydroxyethyl Group

**Figure 2:**
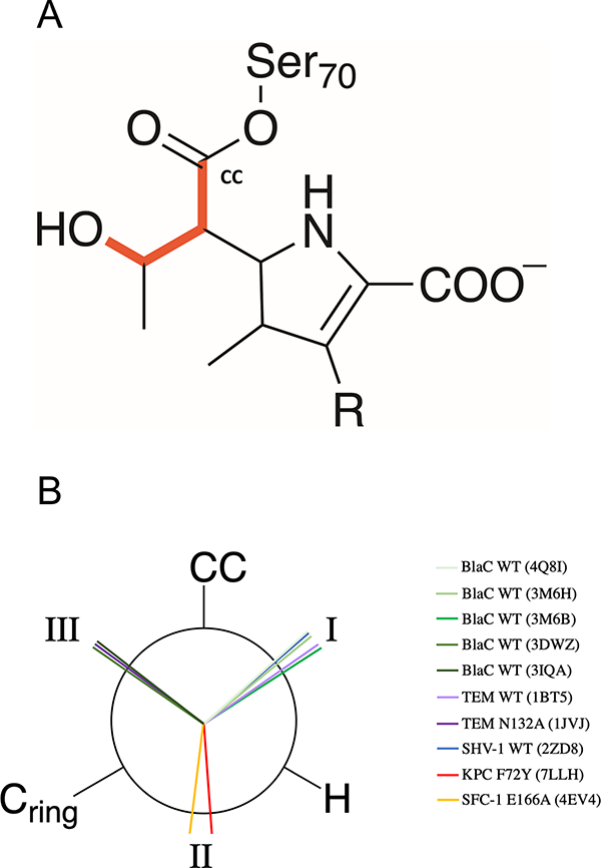
(A)Dihedral measured to describe the rotation of the hydroxyethyl group in the class A β-lactamases with carbapenem antibiotics, relative to the carbonyl carbon (CC). (B) Newman projection showing the relative position of the hydroxyl component of the 6α-1R-hydroxyethyl group in carbapenem acyl-enzyme crystal structures of β-lactamases considered in this study that have been deposited in the PDB. Dihedral angle values can be found in Table S3, while Figure S2 shows representative acyl-enzyme complex structures in each dihedral position.

The rotamer position of the carbapenem 6α-1R-hydroxyethyl group in the β-lactamase acyl-enzyme has been previously suggested to be one of the main differences between carbapenemase enzymes and those that are inhibited by carbapenems.^38,45,46^ Crystal structures of class A carbapenem acyl-enzymes show various conformations of this group (summarized in Figure 2). Three main conformations of the hydroxyethyl group are observed, with dihedral values (between atoms C7, C6, the methyl carbon of the C6 hydroxyethyl group, and the hydroxyl oxygen of the C6 hydroxyethyl group, Figure 2) of approximately 50, 200, and 290°, referred to here as Positions I, II, and III, respectively. These three conformations involve different interactions within the active site. In Position I, the hydroxyl of the 6α-1R-hydroxyethyl group forms a direct hydrogen bond with the DW. In Position II, the methyl group is directed toward DW and Glu166, and the hydroxyl group is rotated away from the active site, hydrogen bonding with the -NH_2_ of the Asn132 sidechain. In Position III, the hydroxyl group is close to Glu166 and DW, but hydrogen bonds only with Oδ1 of Asn132.

**Figure 3:**
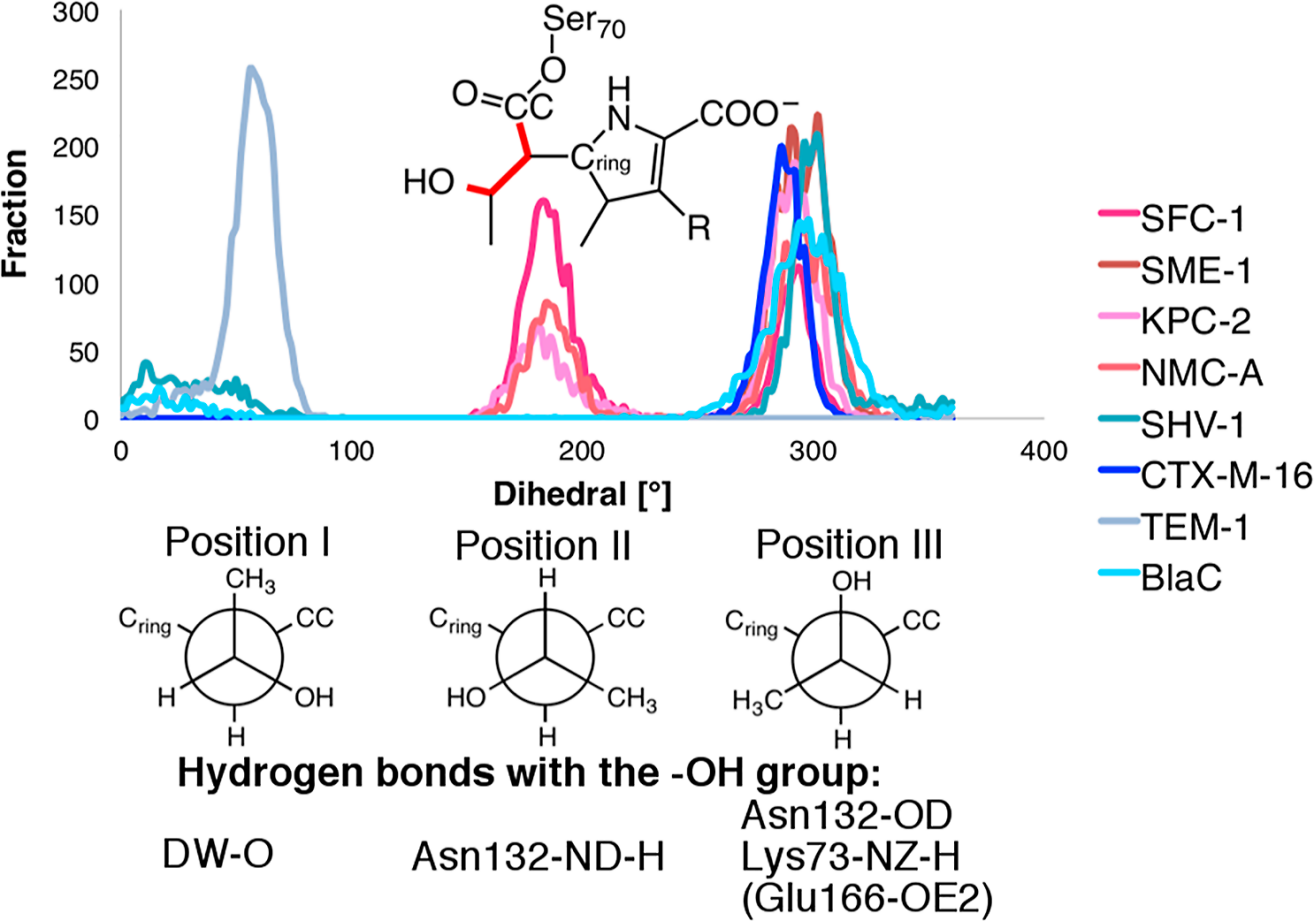
Conformations of the 6α-1R-hydroxyethyl group in class A β-lactamase/meropenem acyl-enzymes. Top: Histogram of the dihedral angles from 3 independent 1ns QM/MM molecular dynamics simulations of all 8 enzymes. Bottom: Schematic indication of the three dihedral positions of the 6α-1R-hydroxyethyl group, including common hydrogen bond interactions of the hydroxyl (−OH) group. CC: carbonyl carbon and C_ring_: carbon from the five-membered ring.

The conformational dynamics of the hydroxyethyl group over time were explored during repeated independent 1 ns unrestrained QM/MM MD simulations of each of the eight meropenem acyl-enzymes. Those simulations showed that the hydroxyethyl group occupies different positions in carbapenem-inhibited enzymes (TEM-1, SHV-1, CTX-M-16, and BlaC) from that in carbapenemases (SME-1, SFC-1, KPC-2, and NMC-A), that is, Positions I and II, respectively (Figure 3). Position III occurs for both carbapenemases and inhibited enzymes. Although Position II was also observed in one of the simulations of CTX-M-16, structural analysis showed that the methyl component of the 6α-1R-hydroxyethyl group replaces DW in the active site (moving DW > 5 Å from its original position). This trajectory, due to the resulting absence of the nucleophile in the active site, is, therefore, a hydrolytically inactive complex and was consequently not included in Figure 3.

The influence of the hydroxyethyl group on activation free energy was also investigated, which, for some systems, required restraining the dihedral to positions not observed in X-ray structures or QM/MM MD simulations. Three enzymes were chosen as representatives of slightly different class A β-lactamase active site arrangements: SFC-1 (carbapenemase), TEM-1 (carbapenem-inhibited), and BlaC (Asn132 substituted by Gly). Umbrella sampling QM/MM MD simulations of the reaction were performed as described above but with a restraint on the hydroxyethyl group dihedral to maintain it in position I, II, or III. Results for these simulations are also presented in Table 1. For the TEM-1/meropenem complex, Position I dominated both in QM/MM MD simulations of the acyl-enzyme and the reaction. The barrier for this system is not affected by applying a restraint to keep the dihedral in position I (17.3 kcal/mol compared to an average of 17.1 kcal/mol without restraint), this is as expected because the dihedral adopts this conformation without restraints. For the carbapenemase SFC-1 (and indeed also for KPC-2 and NMC-A), both positions II and III are observed (Figure 3). When restraints are applied to the position, a slightly higher barrier is observed with the dihedral in position III, whereas a decrease in barrier by 3 kcal/mol (compared to an unrestrained simulation) is observed with the group in position II. Reaction simulations for TEM-1 with a restraint holding the group in Position II were also performed and showed an increased activation barrier (by 2 kcal/mol). For BlaC, reaction simulations without dihedral restraints and with the dihedral restrained to position II gave similar activation free energies (Table 1). This indicates that, by itself, maintaining the hydroxyethyl group in position II is not sufficient to confer carbapenemase activity.

**Table 1:** Calculated Free Energy Barriers for Meropenem Deacylation in the Class a β-lactamases SFC-1, BlaC, and TEM-1, Including Mutants and Restraints on the 6α-1R-Hydroxyethyl Group. All Free Energy Barriers Were Calculated at the SCC-DFTB/ff12SB Level Using Umbrella Sampling and the Weighted Histogram Analysis Method^47^

enzyme	calculated free energy barrier (kcal/mol)^a^
**SFC-1WT(no restraints)**	10.9^b^
**SFC-1WT position III**	10.5
**SFC-1WT position II**	7.7
**SFC-1Cys238Gly position II**	13.8
**SFC-1Asn132Gly(no restraints)**	12.5
**SFC-1Asn132Gly position II**	14.1
**SFC-1Asn132Gly position III**	15.2
**SFC-1 Asn132Gly/Cys238Gly (no restraints)**	16.2
**BlaC WT(no restraints)**	17.9^b^
**BlaC position II**	17.5
**BlaC Gly132Asn(no restraints)**	18.2
**BlaC Gly132Asn position II(no restraints)**	15.7
**BlaC Gly132Asn position II**	14.9
**TEM-1WT(no restraints)**	17.1^b^
**TEM-1WT position I**	17.3
**TEM-1WT position II**	19.1
**TEM-1Asn132Gly(no restraints)**	16.4
**TEM-1Asn132Gly position I**	17.8
**TEM-1Asn132Gly position II**	25.2

a Dihedral restrained to initial position (unless stated otherwise) with a harmonic force constant of 100 kcal mol^–1^ Å^–2^.

b Data taken from Chudyk et al. 2014.^40^

**Figure 4:**
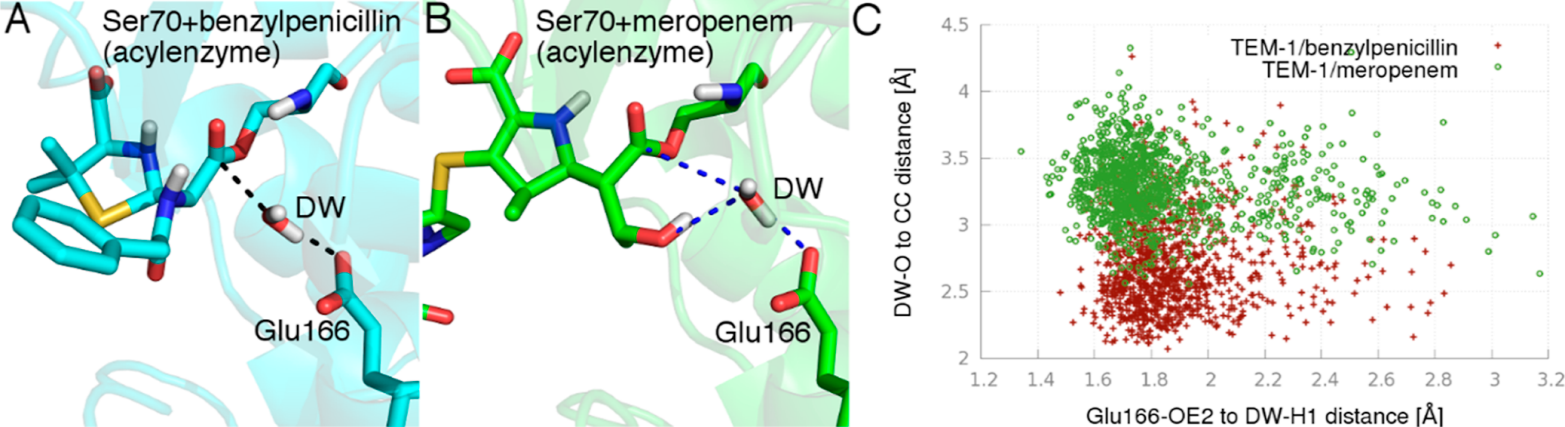
Effect of the 6α-1R-hydroxyethyl group on the nucleophilic attack reaction coordinate in (A) TEM-1/benzylpenicillin; and (B) TEM-1/meropenem acyl-enzymes. The 6α-1R-hydroxyethyl group forms a direct hydrogen bond with the DW. This additional interaction displaces the DW, increasing the distance between the DW and CC (Figure 3) of meropenem compared to the benzylpenicillin acyl-enzyme (C); values are obtained from three independent 1 ns QM/MM MD simulations of each system.

The main structural difference between benzylpenicillin and meropenem that is likely to affect mechanistically important interactions within the β-lactamase active site is the presence of the C6 benzamido and 6α-1R-hydroxyethyl groups, respectively. Although the benzamido group of benzylpenicillin is much larger than the 6α-1R-hydroxyethyl group of meropenem, it does not form any direct hydrogen bonds with the DW or Glu166 and is oriented away from the immediate reaction center. In contrast, the hydroxyl of the 6α-1R-hydroxyethyl group is able to form a direct hydrogen bond with the DW (Figure 4), which has been suggested to influence its nucleophilic properties.^38,45,46^ To further investigate the possible importance of interactions involving the carbapenem 6α-1R-hydroxyethyl group, the behavior of the DW was compared in simulations of the TEM-1 benzypenicilin and meropenem acyl-enzymes. A hydrogen bond to the DW donated by the carbapenem 6α-1R-hydroxyethyl group (in position I) influences the distance between the DW and antibiotic carbonyl carbon (nucleophilic attack reaction coordinate). As shown in Figure 4C, unrestrained QM/MM MD simulations find the acyl-enzyme minimum distances at values of 2.5 and 3.5 Å for the TEM-1 benzylpenicillin and meropenem complexes, respectively. The longer nucleophilic attack distance probably influences the barrier for deacylation: the DW is closer to the carbonyl carbon in the more reactive acyl-enzyme (benzylpenicillin). Based on the free energy surfaces (Figure 1A,C), bringing the DW to around 2.5 Å in the meropenem complex costs ∼5 kcal/mol. The remaining difference between TEM-1/benzylpenicillin and TEM-1/meropenem activity (∼2.5 kcal/mol) may be related to the reduced nucleophilicity of the DW due to its acceptance of a H-bond from the carbapenem 6α-1R-hydroxyethyl group. This situation is similar to what we recently reported for carbapenem hydrolysis by the Class D enzyme OXA-48: a higher barrier for deacylation of OXA-48/meropenem compared to OXA-48/imipenem is related to the 6α-1R-hydroxyethyl group (in position I) donating a H-bond to DW. When the 6α-1R-hydroxyethyl group instead accepts a H-bond from the DW (as observed with OXA-48/imipenem), hydrolysis is more efficient.^48^

### Role of Asn132

**Figure 5:**
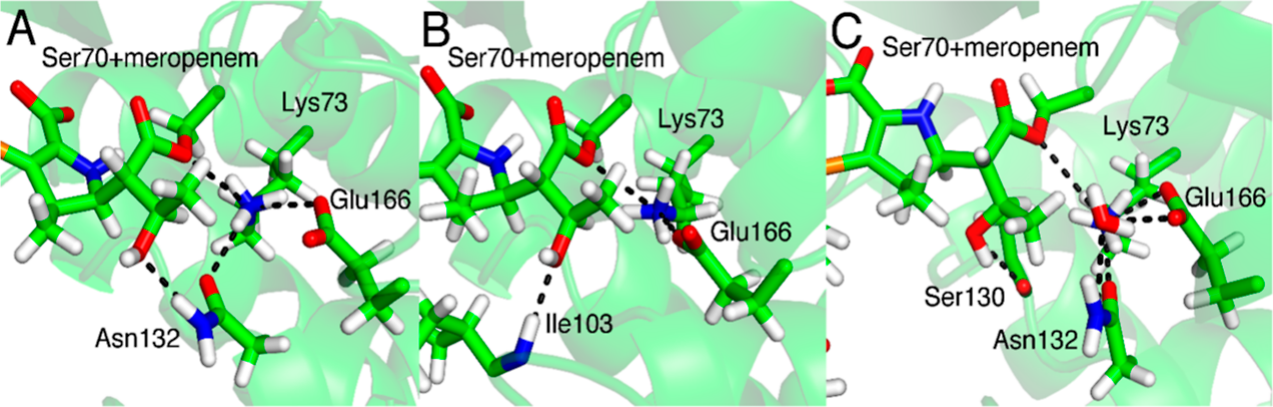
Hydrogen bond network involving the hydroxyl of the 6-1R-hydroxyethyl group in Position II in (A) WT SFC-1, (B) WT BlaC, and (C) WT TEM-1 meropenem acyl-enzymes. Lys73, Glu166, and Asn132/Ile103 are the crucial members of this network. Representative structures from simulations are shown. The hydrogen bonds found are shown with black dotted lines.

The most significant difference between BlaC and other class A β-lactamase active sites is the lack of Asn132 in the former, in which it is replaced by Gly.^49,50^ Asn132 may help keep the 6α-1R-hydroxyethyl dihedral in Position II (Figure 5), thereby lowering the barriers to reaction in carbapenemases. Previous structural and computational studies have suggested that interactions of Asn132 with the 6α-1R-hydroxyethyl group are important in determining carbapenemase activity.^38,43,51,52^ Comparisons between enzymes here (e.g. SFC-1 and TEM-1), show that this residue is less flexible in carbapenem-inhibited enzymes than carbapenemases, due to increased interaction between Asn132 and the 6α-1R-hydroxyethyl in the carbapenem hydrolyzing enzymes. The effects of Asn132 were, therefore, investigated, using QM/MM MD, for three mutated systems with meropenem, SFC-1 Asn132Gly, BlaC Gly132Asn, and TEM-1 Asn132Gly, and compared with the wild-type enzymes.

SFC-1, BlaC, and TEM-1 carrying mutations at position 132 showed significantly different conformational dynamics of the 6α-1R-hydroxyethyl group compared to the respective wild-type enzymes (Figure S3). For the SFC-1 Asn132Gly mutant, Position III of the dihedral dominates. Position II, the dominant position in the wild type enzyme (∼66% of the simulation time), is only present for about 2.5% of the time in simulations of the mutant enzyme. This is due to the loss of the hydrogen bond between Asn132-ND2 and the hydroxyl moiety of the 6α-1R-hydroxyethyl group. For the BlaC Gly132Asn mutant, the dihedral in Position II is observed for a considerable amount of simulation time (about the same time as for Position III (Figure S3), whereas this orientation was not observed in the wild-type acyl-enzyme. In simulations of TEM-1, while Position I dominates the conformational distribution for the wild-type enzyme, the Asn132Gly mutant is able to sample all three positions during 3 ns of MD simulation of the meropenem acyl-enzyme. In Position II, however, the methyl of the 6α-1R-hydroxyethyl group displaces the DW from the active site (as also observed for CTX-M-16, see above and Figure 5), and this trajectory can thus be considered inactive.

As the dihedral occupies Position II for a significant amount of the duration of unrestrained MD simulations of the BlaC Gly132Asn mutant, the possibility of hydrogen bond formation between the meropenem 6α-1R-hydroxyethyl group and Asn132 (as observed for SFC-1) was investigated. In a 1 ns trajectory, where the dihedral is permanently in Position II (Figure 3), a hydrogen bond between the 6α-1R-hydroxylethyl hydroxyl group and ND2 of Asn132 was occupied for 45% of the simulation time. Two different conformations of the Asn132 side chain were identified (Figure S4), with the highest occupied conformation similar to that observed in the crystal structure of the SFC-1 meropenem acyl-enzyme, and the other similar to that observed in profiles obtained for wild-type TEM-1 when the 6α-1R-hydroxyethyl group is restrained to position II.

Free energy surfaces were calculated for the three mutant enzymes to investigate the effect of Asn132 on the energy barrier for meropenem deacylation in SFC-1, TEM-1, and BlaC. The loss of Asn132 increased the barrier for SFC-1 from 10.5 to 12.5 kcal/mol. For the BlaC Gly132Asn mutant, the energy barrier is similar to the wild-type (18.2 vs. 17.9 kcal/mol). However, when the 6-1R-hydroxyethyl group is restrained to position II, the barrier for the mutant enzyme drops to 15.7 kcal/mol. A further reduction (to 14.9 kcal/mol) is observed when a hydrogen bond between Asn132-ND2 and the 6α-1R-hydroxyl is enforced. For the TEM-1 Asn132Gly mutant, when the 6-1R-hydroxyethyl group is restrained to Position I (as observed in the wild-type) the barrier is only slightly higher than in the wild type (17.8 vs 17.1 kcal/mol). However, the absence of Asn132 lowers the activation energy barrier to 16.4 kcal/mol when the 6α-hydroxyethyl group dihedral is unrestrained and can exchange conformations between Positions I and III. Notably, the barrier increases significantly for TEM-1 Asn132Gly (to 25.2 kcal/mol) when the dihedral is restrained to Position II.

### Role of the Oxyanion Hole and Disulfide Bridge

The oxyanion hole and Asn170, which are located on opposite sides of the active site, are responsible for the stabilization of the groups that change formal charge during the reaction: that is, the carbonyl oxygen and Glu166 (See Scheme 1). During the reaction, the negative charge is transferred from the carboxyl group of Glu166 to the DW, and then onto the carbonyl oxygen when the TI is formed. For this reason, the residues of the oxyanion hole (the backbone amide NH groups of Ser70 and Ala/Ser/Thr237) are expected to stabilize the TI relative to the acyl-enzyme (and thereby lower the barrier for deacylation).^44,51,53,54^ Asn170 is expected to have the opposite effect: it stabilizes the deprotonated state of Glu166 (present in the acyl-enzyme) by donating a hydrogen bond.

**Figure 6:**
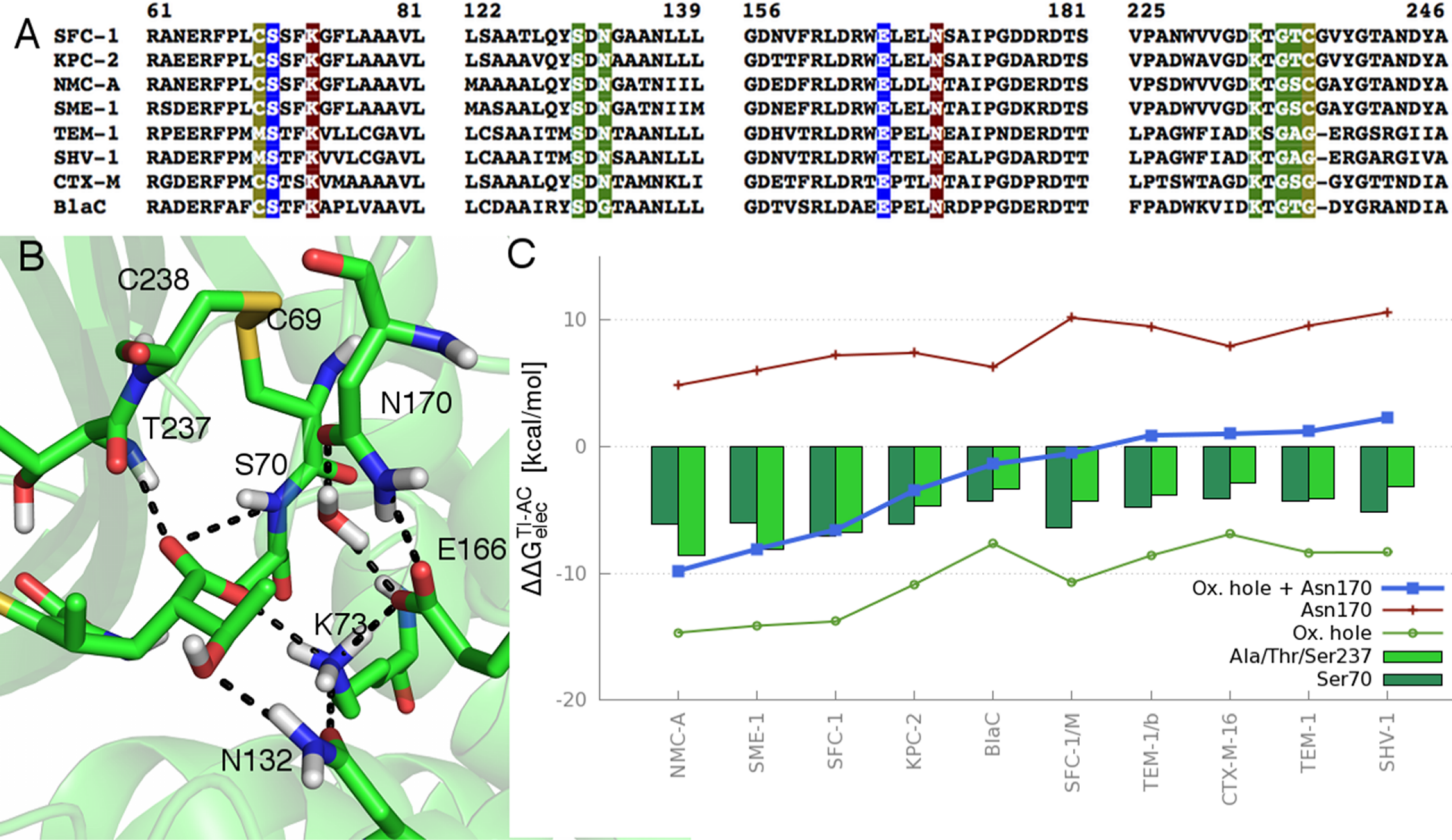
Comparison of features relevant for carbapenem deacylation in different class A β-lactamases. (A) Sequence alignment indicating conservation of active site residues with the following highlights: blue for residues directly involved in the reaction (Ser70 and Glu166), green for those involved in promoting deacylation, and brown for those that disfavor deacylation (by stabilizing the acyl-enzyme). (B) Important residues and their interactions (black dotted lines) in the SFC-1-meropenem acyl-enzyme. (C) Calculated electrostatic stabilization of TI relative to acyl-enzyme of active site components (ΔΔ*G*_elec_^TI–AC^). Oxyanion hole: green line (separate components shown in dark and light green bars); Asn170: red line. TEM-1/b denotes TEM-1 with benzylpenicillin; SFC-1/M denotes the SFC-1 Cys238Gly mutant. Values can be found in Table S3.

The oxyanion hole stabilizes the negative charge concentrated on the carbonyl oxygen during the nucleophilic attack (Figure 6). We quantify this stabilization by comparison of electrostatic interactions between the oxyanion hole and the reacting β-lactam in the acyl-enzyme and the TI (Figure 6C). The stabilization of the TI by the oxyanion hole is stronger in carbapenemases than in carbapenem-inhibited enzymes. This is primarily due to different contributions of Ala/Ser/Thr237, which contribute similarly to Ser70 in carbapenemases, but significantly less in the carbapenem-inhibited class A enzymes.

A significant structural difference between carbapenemases and inhibited enzymes investigated here is the disulfide bond between Cys69 and Cys238 (Figure S1). This disulfide bond constrains the protein backbone around the oxyanion hole and, therefore, may influence its stabilization of the TI.^44^ This disulfide has been identified as potentially essential for the carbapenemase activity of SME-1, and mutations that disrupt the disulfide bond affect enzyme stability in both NMC-A and SFC-1 carbapenemases.^42,55^ However, the disulfide itself does not confer carbapenem-hydrolyzing activity upon all Class A enzymes, see for example GES-5.^42,52,56^ To investigate its role in TI stabilization, the SFC-1 Cys238Gly mutant was constructed (Gly238 is observed in the carbapenem-inhibited enzyme BlaC). Reaction simulations were carried out for the SFC-1 Cys238Gly mutant, with the 6-hydroxyethyl dihedral restrained to Position II (see next section), enabling a direct comparison with wild-type SFC-1 in which this group adopts the same orientation. The calculated barrier for SFC-1 Cys238Gly is 13.5 kcal/mol, 5.4 kcal/mol higher than for the wild-type enzyme, confirming the importance of the disulfide bond in modulating TI stabilization.

Comparison of the electrostatic stabilization by individual residues in SFC-1 Cys238Gly and wild-type SFC-1, and other class A β-lactamases, reveals that the largest differences lie in the contributions of components of the oxyanion hole (Figure 6C), and of Thr237 in particular (TI stabilization by Thr237 drops from −6.8 to −4.3 kcal/mol between wild-type SFC-1 and SFC-1 Cys238Gly). A small decrease was also observed for the Ser70 amide (from −7.0 to −6.4 kcal/mol). Together, these changes make SFC-1 Cys238Gly similar to the carbapenem-inhibited enzymes with regard to interactions involving the oxyanion hole. The lack of the disulfide bridge in the Cys238Gly SFC-1 (and in carbapenem-inhibited enzymes) causes a slight relaxation of the structure around the active site, increasing the distance between the Thr237 backbone amide and the carbonyl oxygen. However, in none of the simulations of any of the enzymes did the carbonyl oxygen of meropenem move out of the oxyanion hole.

### Role of Asn170

Electrostatic calculations also confirm that Asn170 has a destabilizing effect on TI formation (i.e., increasing the barrier of reaction) for all the class A β-lactamases studied here (Figure 6C). This destabilizing effect is weaker in carbapenemases than in carbapenem-inhibited enzymes, however, with a difference of up to 5 kcal/mol (between NMC-A and most carbapenem-inhibited enzymes). This difference can be explained by a slight movement of Asn170 away from Glu166 and DW in carbapenemases relative to its position in carbapenem-inhibited enzymes; this movement is apparent from crystal structures.^38^ In addition, this repositioning of Asn170 allows the 6α-1R-hydroxyethyl group to more readily adopt dihedral Position II, by increasing the active site volume and thus preventing steric clashes with the 6α-1R-hydroxyethyl group that would otherwise occur.

## Discussion

There is an urgent need to understand the molecular determinants of growing bacterial resistance to carbapenems caused by β-lactamases. Identification of factors contributing to carbapenemase activity may facilitate understanding of the basis for resistance mediated by such enzymes, and, therefore, provide a basis for antibiotic (re)design in order to overcome this growing clinical problem. Crystal structures in the PDB^38,43,46,57−61^ provide insight into active site interactions in the acyl-enzyme state, but the details of why some enzymes are able to efficiently hydrolyze carbapenems, rather than forming stable acyl-enzymes, are unclear. Answering this question requires an analysis of the reactivity of the acyl-enzymes.

Here, we have used multiscale simulations to model the rate-limiting first step of the carbapenem deacylation reaction for a panel of class A β-lactamases that differ in activity toward these important antibiotics. The QM/MM simulations correctly discriminate between carbapenemases and enzymes that are inhibited by carbapenems. The results help explain why some class A β-lactamases readily hydrolyze carbapenem antibiotics, whereas other, very similar, enzymes do not. Several subtle structural effects, not evident from visual inspection of either the active sites of carbapenemases or of the acyl-enzyme complexes, are identified from our QM/MM simulations. Electrostatic interactions dominate catalysis in enzymes.^62−64^ For the β-lactamases studied here, electrostatic interactions within their active sites were analyzed, several important residues that affect the activity of the enzymes studied were identified, and their effects on the TI were studied. These include components of the oxyanion hole (backbone amide NH groups of Ala/Ser/Thr237 and Ser70), the adjacent disulfide bridge, as well as Asn170, Lys73, and Asn132. Interactions involving the carbapenem 6α-1R-hydroxyethyl group, and its rotameric position, were also of considerable importance, exerting their effects upon the Glu166 general base and the DW molecule as well as on the residues mentioned above.

We performed further QM/MM simulations of mutants, and also enforced specific conformations, to test the effects on the reaction barrier. It has been previously suggested that the carbapenem 6α-1R-hydroxyethyl group can retard the deacylation rate by forming H-bonds with the DW, which weakens its nucleophilicity.^61,65^ Our simulations indicate that the 6α-1R-hydroxyethyl group being oriented in positions I or III (seen in carbapenem-inhibited enzymes) leads to a higher deacylation barrier. These positions somewhat displace the DW, preventing efficient nucleophilic attack upon the acyl-enzyme carbonyl carbon. Of the three distinct rotamers for the carbapenem 6α-1R-hydroxyethyl group, only position II allows the polar component (the hydroxyl group) to point out of the binding site, leading to lower reaction barriers. Position II is observed, both in X-ray structures and during our QM/MM MD calculations, only for carbapenem complexes of carbapenem-hydrolyzing enzymes.^38,57^ We further note the possibility that elimination of the hydroxyethyl group may, under some circumstances, occur on reaction with class A beta-lactamases. This would diminish the unfavorable hydroxyethyl-DW interactions seen in carbapenem-inhibited enzymes; although it has not been widely reported and is yet to be observed crystallographically.^66^

Close contacts between the hydroxyethyl group and Asn132 and/or DW were also observed in our simulations. In carbapenemases, the Asn132 side chain can form a hydrogen bond to the carbapenem hydroxyethyl group, keeping it in the (carbapenemase-specific) Position II. The importance of avoiding steric clashes involving the hydroxyethyl group was suggested by investigations of the TEM-1 Asn132Ala mutant (PDB ID 1JVJ).^61^ Removal of the Asn132 side chain led to less strained interactions within the carbapenem binding site and a different orientation of bound imipenem with respect to the oxyanion hole (with the carbonyl oxygen pointing into the oxyanion hole, as opposed to its position in the X-ray structure of the complex with the wild-type enzyme^46^). Positional shifts of Asn132 observed in various crystal structures, as well as the simulations performed here of the Asn132Gly substitution in BlaC, support the importance of residue 132, which indirectly influences reaction barriers.^67,68^

It has been suggested that the stronger stabilization effect of the oxyanion hole in carbapenemases relates to the Cys69-Cys238 disulfide bridge located just behind the oxyanion hole.^31,42^ Indeed, the SME-1 Cys69Ala mutant shows a lack of imipenemase activity.^39,67^ In our simulations, the SFC-1 Cys69Gly mutant has a higher barrier (14.1 kcal/mol), associated with weaker TI interactions with the oxyanion hole, and a stronger destabilizing effect of Asn170 on the TI than was found for wild-type SFC-1. This highlights the importance of the Cys69-Cys238 disulfide bridge for carbapenemase activity: disruption of this places SFC-1 Cys69Gly above the ∼13 kcal mol^–1^ activation energy barrier discriminator for carbapenemase activity.^40,41^

The destabilization of the TI relative to the acyl-enzyme by Asn170 is due to its coordination of the DW and Glu166 in the acyl-enzyme: Asn170 forms two hydrogen bonds with the DW and Glu166. This stabilizes the carboxylate form of Glu166 but lowers the nucleophilicity of the DW similarly to interactions involving the hydroxyethyl group.^65,69^ The increased destabilization of the TI compared to the acyl-enzyme by Asn170 correlates well with the calculated free energy barriers. Asn170 contributes the least to the destabilization of the TI in NMC-A, which has both the lowest (calculated and experimentally determined) free energy barrier to deacylation. All carbapenem-inhibited enzymes other than BlaC (including SFC-1 Cys238Gly) experience greater destabilization of the TI by Asn170 compared to carbapenemase enzymes, indicating the importance of Asn170 destabilization of the TI in inhibiting carbapenemase activity. Furthermore, kinetic and structural studies on GES-type class A β-lactamases, comparing Asn170 in GES-2 with Gly170 and Ser170 in GES-1 and GES-5, respectively, in the context of otherwise identical active sites, also show the role of Asn170 in coordinating the DW.^52^ It has been suggested that the formation of a carbapenem acyl-enzyme complex with GES-1 and -5 causes a displacement of the DW, suggesting that a water molecule from bulk solvent would have to enter the active site to facilitate deacylation. In contrast, the DW remains in the GES-2 active site due to an additional interaction with the Asn170 side chain, not present in GES-1 or -5. This interaction, however, reduces the nucleophilicity of the DW, attenuating the nucleophilic attack on the acyl-enzyme complex. The result is that GES-2 displays poor, but measurable, carbapenemase activity. GES-5, despite requiring a bulk solvent molecule to enter the active site and take the position of the displaced DW, does not attenuate the nucleophilic attack because a serine rather than an asparagine is present at position 170. This results in deacylation being more efficient in GES-5 than GES-2.^52,70^ Alternatively, an N170A substitution in KPC-2 significantly reduces the carbapenemase activity of the enzyme.^30^ Clearly, the balance between maintaining a DW in the active site to participate in deacylation and reducing its nucleophilicity is subtle and can significantly affect β-lactam breakdown efficiency, as is also the case in the OXA-48-like β-lactamases, and as has been shown in KPC-2 variants.^30,71,72^ As these results show, simulations of reactivity (e.g. with QM/MM methods) can play a crucial part in identifying determinants of activity.

## Conclusions

Our results explain the differences between class A β-lactamases that are efficient carbapenemases and those that are inhibited by carbapenems. The results point to a combination of synergistic interactions conferring carbapenemase activity. The combination of multiple, subtle, changes to active site structure and dynamics results in the enhanced carbapenem hydrolysis rates of carbapenemases. Important factors include the oxyanion hole, the Cys69-Cys238 disulfide bridge, interactions involving Asn132 and Asn170, as well as changes in the orientation of the 6α-1R-hydroxyethyl group of the carbapenem scaffold. Our simulations indicate that, while disruption of any single contributor can lead to loss of carbapenemase activity in an enzyme such as SFC-1, individual factors (constraining the carbapenem hydroxyethyl group into an orientation associated with hydrolysis, or introduction of Asn132 into the BlaC enzyme) are, on their own, insufficient to confer carbapenemase activity on an inactive scaffold. Exploiting the interactions identified here may provide direction for routes to develop new β-lactam antibiotics able to evade the activity of class A carbapenemases, and, thus ultimately present new treatment options for infections by resistant Gram-negative bacteria.

## Methods

### Construction of Hydrolysis Models and Free Energy Calculations

The protein/ligand complexes in the acyl-enzyme state were prepared based on the respective crystal structures following protocols described previously.^40^ The following β-lactamases were set up for QM/MM calculations: SFC-1, KPC-2, NMC-A, SME-1, TEM-1, SHV-1, BlaC and CTX-M-16, the single mutants SFC-1 Asn132Gly, BlaC Gly132Asn, SFC-1 Cys238Gly, TEM-1 Asn132Gly and the double mutant SFC-1 Asn132Gly/Cys238Gly. TEM-1 was prepared with acyl-enzymes of two different ligands, benzylpenicillin and meropenem, and the other β-lactamases were set up as meropenem acyl-enzyme complexes. The AMBER ff12SB force field^73−75^ was used for all calculations, as implemented in the AMBER12 software package.^74,75^

The procedure for system setup and parameterization has been described and tested in detail.^40^ In short, meropenem was parametrized using RESP charges and GAFF small molecule parameters. Each protein was prepared according to a similar procedure: the protonation states of ionizable residues were assigned based on calculated p*K*_a_ values, most crystallographic water molecules (apart from the deacylating water) were deleted and the solvent was then added to the system using the AmberTools program tLEaP.^74,75^ Each system was neutralized with appropriate counterions and solvated with a TIP4P water box extending at least 10 Å from any protein atom. The acyl-enzymes were divided into QM and MM regions for QM/MM calculations at the SCC-DFTB/ff12SB level: for systems containing meropenem, the QM region consists of 41 atoms and 3 link atoms. The Glu166 sidechain (from the CG atom), and the DW molecule, were treated at the QM level. The meropenem covalently bound to Ser70 was also included in the QM region, from the CB of Ser70 to the S atom of the meropenem C2 substituent; the remainder of the C2 group is far from the catalytic residues (and mostly exposed to bulk solvent). For the TEM-1 complex with benzylpenicillin, the QM region consisted of 54 atoms and 2 link atoms and included Ser70 from CB onward and the entire antibiotic.

To compare the carbapenemase activity of SFC-1, KPC-2, SME-1, and NMC-A with carbapenem-inhibited TEM-1, SHV-1, CTX-M-16, and BlaC enzymes, we simulated the rate-limiting first step of deacylation^29^ of the acyl-enzymes formed on reaction of these eight class A β-lactamases with the carbapenem meropenem using the QM/MM (SCC-DFTB/ff12SB) umbrella sampling QM/MM method. For comparison, deacylation of the acyl-enzyme of TEM-1 with the good substrate benzylpenicillin was also simulated.

A standard QM/MM equilibration protocol^40,41^ was used in all cases, including minimization, heating in the *NVT* ensemble, and QM/MM MD in the NPT ensemble. 300 ps unrestrained SCC-DFTB/ff12SB MD was carried out prior to reaction calculations. In addition, three independent 1 ns QM/MM MD simulations per system were performed to investigate interactions in the acyl-enzyme state.

The first step of deacylation was investigated by calculating a 2D free energy surface. 20 ps of MD was run at each window, resulting in 4.1 and 7.5 ns of QM/MM MD for a single surface of the benzylpenicillin and meropenem reaction simulations, respectively. The reaction coordinates used to model the proton transfer and TI formation (nucleophilic attack) are shown in Chart S1. All reaction simulations were performed in the forward direction, starting with the acyl-enzyme, and finishing with the TI. The inclusion of harmonic distance restraints with a force constant of 100 kcal mol^–1^ A^–2^) led to umbrella sampling windows with good overlap. The weighted histogram analysis method (WHAM)^47,76^ was used to calculate the potential of mean force. Reaction simulations were also performed with harmonic distance restraints (with a force constant of 100 kcal mol^–1^ A^–2^) between the 6α-1R-hydroxyethyl group oxygen and Asn132 ND2, which were carried out to keep this group in a specific orientation (target values of 50, 200, or 290°) (Figure 3).

### Electrostatic Contributions of Active Site Amino Acids to Catalysis

To analyze the differences in catalytic activity between the various class A β-lactamases, the contribution to the stabilization of every active site residue in the reaction was evaluated. As shown previously, electrostatics is central in biological catalysis.^63,77,78^ We analyzed the stabilization of the TI because this high-energy minimum is representative of the two transition states involved in the deacylation reaction (and its formation is believed to be rate-limiting). The stabilizing effect was calculated as the difference in electrostatic interaction energy between a residue and the QM region in the acyl-enzyme (AC) and the TI, using an average of over 1000 snapshots generated during 20 ps of QM/MM MD simulation of each state:
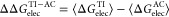
1

A negative value of ΔΔ*G*_elec_^TI–AC^ thus indicates stabilization of the TI with respect to the acyl-enzyme, and vice versa. For these calculations, we considered stabilization of the complete QM region excluding the meropenem C2 substituent beyond the S atom (i.e., the five-membered ring with a negatively charged carboxylate group). The exclusion of the latter avoided noise from the strong interactions between the carboxylate group and the enzyme environment. While these interactions should be essentially identical between the AC and TI, any variations in the trajectories sampled could overshadow the stabilization of atoms actively involved in the reaction. The reaction coordinate values of the compared states were deliberately kept constant (0.8, 3.5 Å) and (−0.8, 1.4 Å) for the AC and TI, respectively, to enable direct comparison between different enzymes/variants. Trajectories for the AC were taken from the reaction simulations. To generate comparable structures, avoiding large structural changes around the active site, trajectories for the TI were generated as follows: (1) minimization of the system starting from the AC endpoint with the new reaction coordinate restraints (−0.8, 1.4 Å) applied, (2) heating from 50 to 300 K in 50 ps (as in ref (40)), and (3) 20 ps of QM/MM umbrella sampling MD at the new reaction coordinate. Mulliken charges for the QM atoms at each snapshot were collected for electrostatic interaction calculations, which were performed with the SIRE software.^79^ Mulliken charges have well-known limitations for describing electrostatic potentials but are useful here as the focus is on the change in charge (and the associated change in interaction) between the two states.

## Supporting Information Available

Progress coordinates of the QM/MM umbrella sampling MD simulations; example structure of the active site of class A serine β-lactamases; structure showing two representative Asn132 conformations from cluster analysis of QM/MM MD simulations; residue interaction energies; 6α-1R-hydroxyethyl group dihedral values of crystal structures (from the PDB) of acyl-enzymes considered in this study; histogram of dihedral values of SFC-1 Asn132Gly, TEM-1 Asn132Gly, and BlaC Gly132Asn from QM/MM umbrella sampling MD simulations (PDF)

## Notes

**Data availability statement:** structures of the acyl-enzyme complex, transition state and tetrahedral intermediate of example enzymes (CTX-M-16, KPC-2, and SME-1) and Input files for all simulations are available at the University of Bristol data.bris Research Data Repository (https://data.bris.ac.uk/data/). Ligand parameter files are available at https://figshare.com/articles/dataset/Carbepenem_acyl-enzyme_parameters_for_beta-lactamase_simulations_Meropenem/8158097.

## Abbreviations

QM/MM: quantum mechanics/molecular mechanics
DW: deacylating water
CDC: Centers for Disease Control and prevention
PBP: penicillin-binding protein
WHAM: weighted histogram analysis method
TI: tetrahedral intermediate
AC: acyl-enzyme
RC: reaction coordinate
