# Supplementary material for: QM/MM Simulations Reveal the Determinants of Carbapenemase Activity in Class A β-Lactamases
Source: ACS Infect Dis. 2022 Jul 25;8(8):1521–32. doi: 10.1021/acsinfecdis.2c00152 (PMC9379904; doi:10.1021/acsinfecdis.2c00152)
Supplement: Supplementary file 1 — id2c00152_si_001.pdf [file id2c00152_si_001.pdf]

Supporting Information

QM/MM Simulations Reveal the Determinants of Carbapenemase Activity in Class A β-lactamases

Ewa I. Chudyk†,‡, Michael Beer¶†, Michael A. L. Limb†, Charlotte A. Jones†, James Spencer¶, Marc W. van der Kamp§†, and Adrian J. Mulholland*†*

†Centre for Computational Chemistry, School of Chemistry, University of Bristol, Cantock's Close, Bristol BS8 1TS, United Kingdom, § School of Biochemistry and ¶ School of Cellular and Molecular Medicine, University of Bristol Medical Sciences Building, University Walk, Bristol BS8 1TD, United Kingdom

*E-mail: Marc.vanderKamp@bristol.ac.uk; Adrian.Mulholland@bristol.ac.uk

Pages: 11, Figures: 4, Tables: 3, Charts: 1

Table of Contents

Experimental and Calculated Free Energy Barriers to Deacylation S3

Reaction Coordinates S4

Representative Structure of Class A β-lactamase Active Site S5

6α-1R-Hydroxyethyl Dihedral Angle Values of Crystal Structures Deposited on the PDB S6

6α-1R-Hydroxyethyl Orientations (Positions I, I & III) Found in Crystal Structures Deposited on the PDB S7

Electronic Decomposition Values for Studied Enzymes S8

6α-1R-Hydroxyethyl Dihedral Angle Frequency Histogram of Mutated SFC-1, BlaC and TEM-1 Systems S9

Asn132 Conformations Derived from Cluster Analysis S10

References..S11

Table S1. Experimental data showing the k_{cat} values for carbapenem hydrolysis in each of the 8 studied enzymes and the apparent free energy barriers derived from these ($\Delta^{\ddagger}G_{exp}$) compared to the calculated free energy barriers ($\Delta^{\ddagger}G_{calc}$). All data from Chudyk *et al.*, 2014(1).

Enzyme	k_{cat} (s^{-1})	$\Delta^{\ddagger}G_{exp}$ (kcal mol^{-1})	$\Delta^{\ddagger}G_{calc}$ (kcal mol^{-1})
BlaC	1.7×10^{-3}	21.5	17.9 (0.08)
CTX-M-16	4.2×10^{-3}	20.8	18.9 (1.09)
SHV-1	1.3×10^{-3}	21.6	17.0 (0.43)
TEM-1	2.3×10^{-3}	22.7	17.1 (0.43)

KPC-2	3.6	16.8	10.5 (0.88)
NMC-A	12.0	16.1	7.5 (0.43)
SFC-1	6.5	16.6	10.9 (0.86)
SME-1	3.2	16.9	10.3 (2.80)

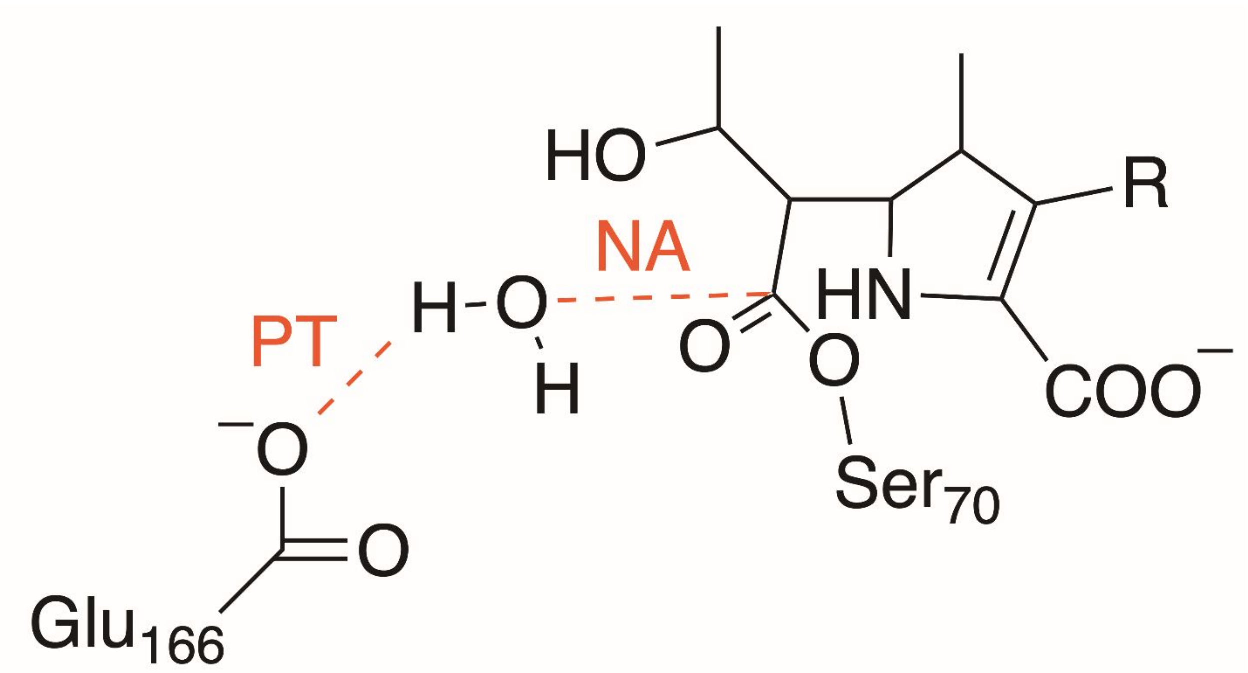

Chart S1: The reaction coordinates used to model the reaction during the QM/MM umbrella sampling simulations. The proton transfer (PT) reaction coordinate measures the distance between a carboxyl oxygen of Glu166 and the transferring proton minus the distance between the transferring proton and the DW oxygen. The nucleophilic attack (NA) coordinate is the distance between the oxygen of DW and carbonyl carbon of meropenem.

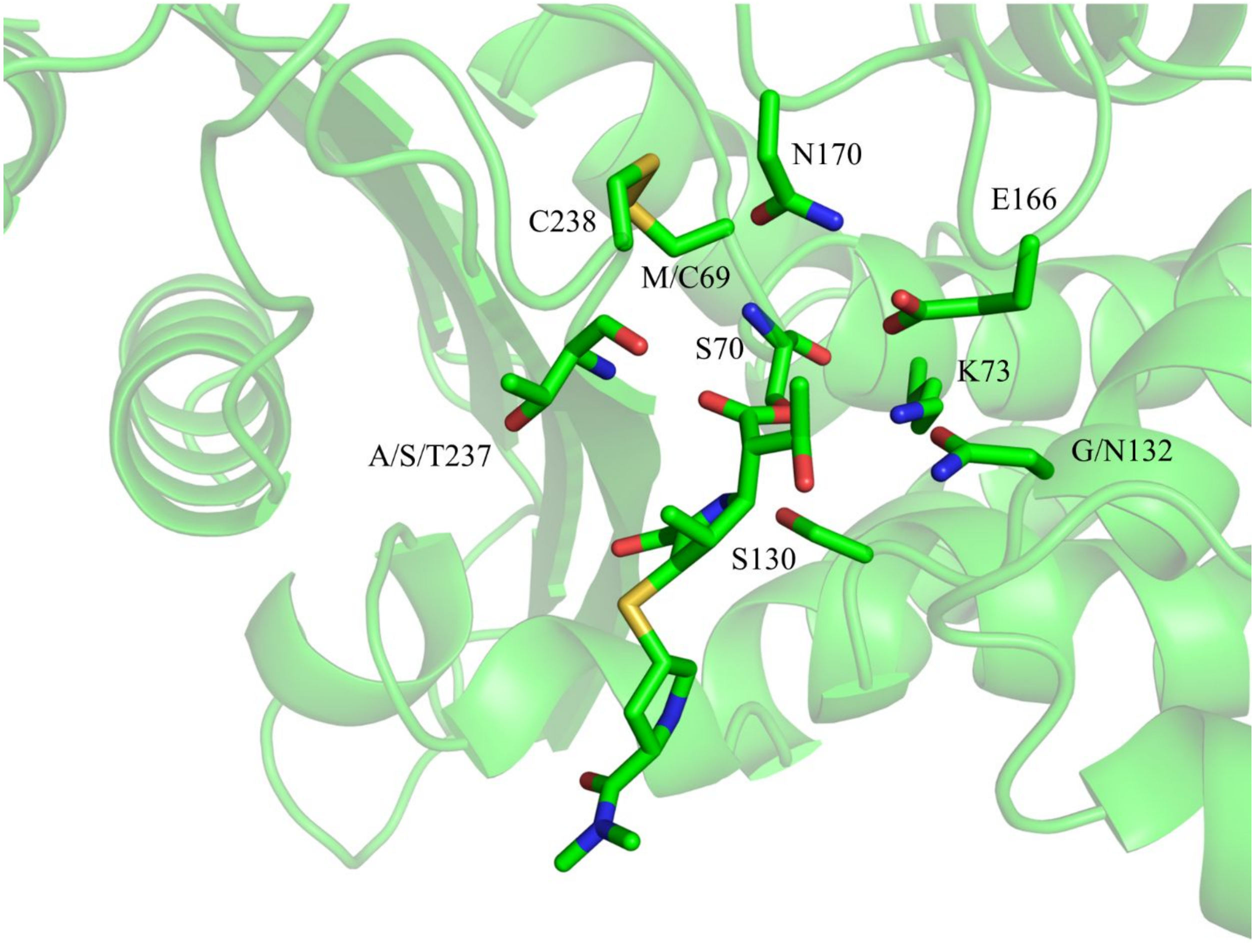

Figure S1. Arrangement of active site residues in class A serine β-lactamases. The KPC-2 active site is shown, taken from a snapshot of the QM/MM 1ns acyl-enzyme complex simulation. The meropenem ligand is left in to show the residue positions relative to the bound carbapenem. Residue 237 and Ser70 backbone is also shown, due to its role in stabilizing the oxyanion present

in the deacylation tetrahedral intermediate. Residues are labelled, including variants found in any of the 8 class A serine β-lactamases studied.

Table S2. Dihedral values of the 6α-1R-hydroxyethyl group from deposited crystal structures on the PDB.

PDB ID	Enzyme	Acylated Carbepenem	Dihedral Value (degrees)	Dihedral Position
1BT5(2)	TEM-1 WT	Imipenem	55	I
1JVJ(3)	TEM-1 N132A Mutant	Imipenem	301	III
2ZD8(4)	SHV-1 WT	Meropenem	45	I
3DWZ(5)	BlaC WT	Meropenem	295	III
3M6B(6)	BlaC WT	Ertapenem	57	I
3M6H(6)	BlaC WT	Ertapenem	51	I
3IQA(6)	BlaC WT	Doripenem	303	III
4Q8I(7)	BlaC WT	Tebipenem	45	I
4EV4(8)	SFC-1 E166A Mutant	Meropenem	194	II
7LLH(9)	KPC-2 F72Y Mutant	Imipenem	174	II

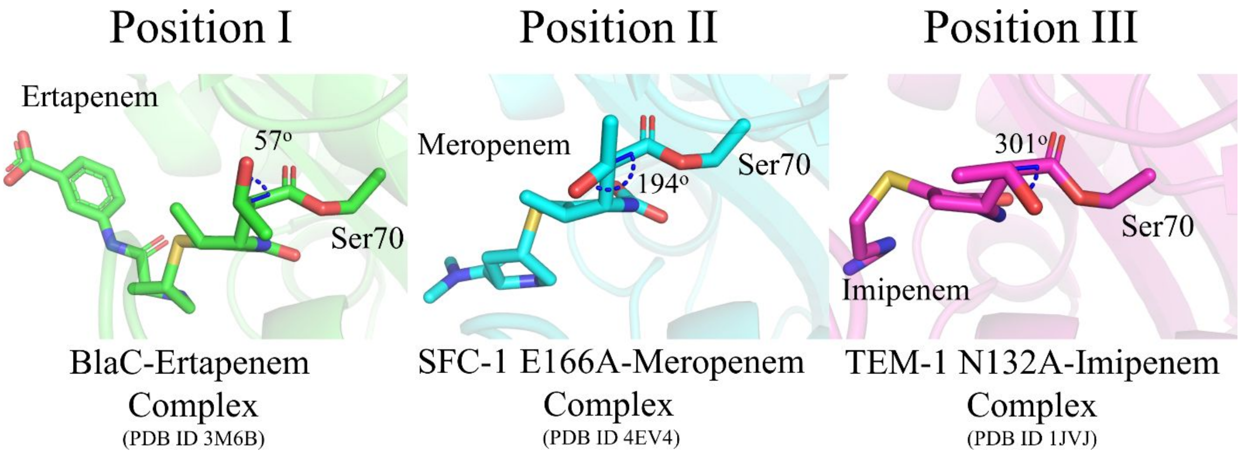

Figure S2. The orientation of the 6α-1R-Hydroxyethyl group in each position. Dihedral angle values (see Table S2) are shown.

Table S3. Electronic decomposition values for each system that underwent the analysis using the SIRE software. Values are shown for the contribution of Ser 70, Ser/Thr 237, Asn 170 and Gly/Asn 132.

**Enzyme**	**Ser 70 (kcal mol^{-1})**	**Ser/Thr 237 (kcal mol^{-1})**	**Asn 170 (kcal mol^{-1})**	**Gly/Asn 132 (kcal mol^{-1})**
**BlaC**	-4.32	-3.35	6.30	-6.98
**CTX-M-16**	-4.10	-2.83	7.94	-3.08

**SHV-1**	-5.19	-3.17	10.62	-5.92
**TEM-1**	-4.28	-4.08	9.56	-4.42
**TEM-1/benzylpenicillin**	-4.80	-3.81	9.51	-3.83
**KPC-2**	-6.13	-4.73	7.42	-1.81
**NMC-A**	-6.11	-8.57	4.87	-9.81
**SFC-1 (Position I)**	-7.04	-6.75	7.22	-1.70
**SFC-1 (Position II)**	-6.94	-6.49	7.41	1.40
**SFC-1 Cys238Gly**	-6.42	-4.29	10.19	-5.97
**SME-1**	-6.02	-8.12	6.04	-7.63

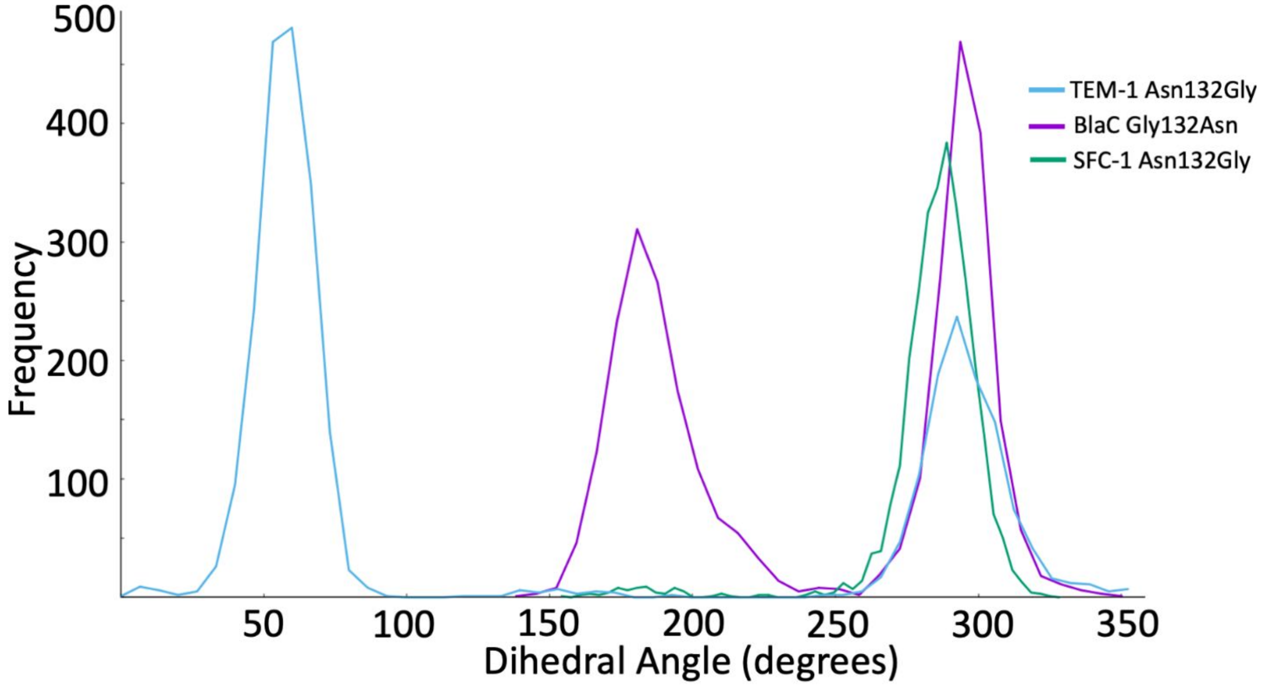

Figure S3. Dihedral angle frequency of mutated systems to understand the role of Asn132 in determining the 6α-1R-hydroxyethyl group conformation. The dihedral angle values shown were calculated from SFC-1 Asn132Gly (Green), BlaC Gly132Asn (Purple) and TEM-1 (Asn132Gly) QM/MM umbrella sampling simulations with no additional restraints on the 6α-1R-hydroxyethyl group.

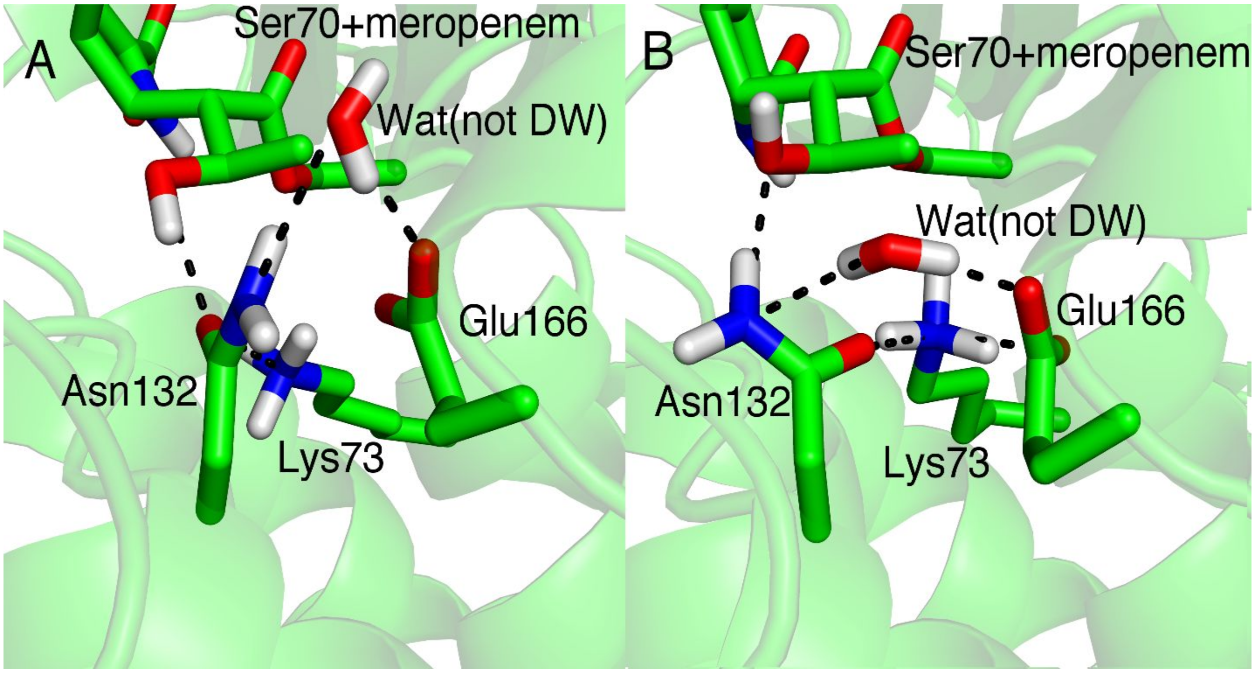

Figure S4: Representative structures of two primary Asn132 conformations obtained from cluster analysis. A) First, highest occupied, conformation (78%) and B) Second conformation (21%).

SUPPORTING INFORMATION REFERENCES

1. Chudyk, E.I., M.A. Limb, C. Jones, J. Spencer, M.W. van der Kamp, and A.J. Mulholland, *QM/MM simulations as an assay for carbapenemase activity in class a beta-lactamases.* Chem Commun, 2014. **50**: p. 14736-14739.
2. Maveyraud, L., L. Mourey, L.P. Kotra, J.-D. Pedelacq, V. Guillet, S. Mobashery, and J.P. Samama, *Structural Basis for Clinical Longevity of Carbapenem Antibiotics in the Face of Challenge by the Common Class A Beta-Lactamases from Antibiotic-Resistant Bacteria.* Journal of the American Chemical Society, 1998. **120**.
3. Wang, X., G. Minasov, and B.K. Shoichet, *Noncovalent interaction energies in covalent complexes: TEM-1 beta-lactamase and beta-lactams.* Proteins, 2002. **47**(1): p. 86-96.
4. Nukaga, M., C.R. Bethel, J.M. Thomson, A.M. Hujer, A. Distler, V.E. Anderson, J.R. Knox, and R.A. Bonomo, *Inhibition of Class A Beta-Lactamases by Carbapenems: Crystallographic Observation of Two Conformations of Meropenem in SHV-1.* J Am Chem Soc, 2008. **130**(38): p. 12656-62.
5. Hugonnet, J.-E., W. Tremblay Lee, I. Boshoff Helena, E. Barry Clifton, and S. Blanchard John, *Meropenem-Clavulanate Is Effective Against Extensively Drug-Resistant Mycobacterium tuberculosis.* Science, 2009. **323**(5918): p. 1215-1218.
6. Tremblay, L.W., F. Fan, and J.S. Blanchard, *Biochemical and Structural Characterization of Mycobacterium tuberculosis β-Lactamase with the Carbapenems Ertapenem and Doripenem.* Biochemistry, 2010. **49**(17): p. 3766-3773.
7. Hazra, S., H. Xu, and J.S. Blanchard, *Tebipenem, a New Carbapenem Antibiotic, Is a Slow Substrate That Inhibits the β-Lactamase from Mycobacterium tuberculosis.* Biochemistry, 2014. **53**(22): p. 3671-3678.
8. Fonseca, F., E.I. Chudyk, M.W. van der Kamp, A. Correia, A.J. Mulholland, and J. Spencer, *The Basis for Carbapenem Hydrolysis by Class A β-Lactamases: A Combined Investigation using Crystallography and Simulations.* Journal of the American Chemical Society, 2012. **134**(44): p. 18275-18285.
9. Furey, I.M., S.C. Mehta, B. Sankaran, L. Hu, B.V.V. Prasad, and T. Palzkill, *Local interactions with the Glu166 base and the conformation of an active site loop play key roles in carbapenem hydrolysis by the KPC-2 β-lactamase.* Journal of Biological Chemistry, 2021. **296**.
